# Efficacy of low carbohydrate and ketogenic diets in treating mood and anxiety disorders: systematic review and implications for clinical practice

**DOI:** 10.1192/bjo.2023.36

**Published:** 2023-04-17

**Authors:** Daniel M. Dietch, Jess Kerr-Gaffney, Meghan Hockey, Wolfgang Marx, Anu Ruusunen, Allan H. Young, Michael Berk, Valeria Mondelli

**Affiliations:** Lonsdale Medical Centre, London, UK; and Department of Psychological Medicine, Institute of Psychiatry, Psychology & Neuroscience, King's College London, London, UK; Department of Psychological Medicine, Institute of Psychiatry, Psychology & Neuroscience, King's College London, London, UK; Institute for Mental and Physical Health and Clinical Translation (IMPACT), Food & Mood Centre, School of Medicine, Barwon Health, Deakin University, Geelong, Australia; Institute for Mental and Physical Health and Clinical Translation (IMPACT), Food & Mood Centre, School of Medicine, Barwon Health, Deakin University, Geelong, Australia; Department of Psychiatry, Kuopio University Hospital, Kuopio, Finland; and Institute of Public Health and Clinical Nutrition, University of Eastern Finland, Kuopio, Finland; Department of Psychological Medicine, Institute of Psychiatry, Psychology & Neuroscience, King's College London, London, UK; and National Institute for Health Research (NIHR) Maudsley Biomedical Research Centre, South London and Maudsley NHS Foundation Trust, King's College London, London, UK

**Keywords:** Ketogenic diet, nutritional psychiatry, mood disorders, low carbohydrate diet, anxiety disorders

## Abstract

**Background:**

There is mounting interest in the potential efficacy of low carbohydrate and very low carbohydrate ketogenic diets in various neurological and psychiatric disorders.

**Aims:**

To conduct a systematic review and narrative synthesis of low carbohydrate and ketogenic diets (LC/KD) in adults with mood and anxiety disorders.

**Method:**

MEDLINE, Embase, PsycINFO and Cochrane databases were systematically searched for articles from inception to 6 September 2022. Studies that included adults with any mood or anxiety disorder treated with a low carbohydrate or ketogenic intervention, reporting effects on mood or anxiety symptoms were eligible for inclusion. PROSPERO registration CRD42019116367.

**Results:**

The search yielded 1377 articles, of which 48 were assessed for full-text eligibility. Twelve heterogeneous studies (stated as ketogenic interventions, albeit with incomplete carbohydrate reporting and measurements of ketosis; diet duration: 2 weeks to 3 years; *n* = 389; age range 19 to 75 years) were included in the final analysis. This included nine case reports, two cohort studies and one observational study. Data quality was variable, with no high-quality evidence identified. Efficacy, adverse effects and discontinuation rates were not systematically reported. There was some evidence for efficacy of ketogenic diets in those with bipolar disorder, schizoaffective disorder and possibly unipolar depression/anxiety. Relapse after discontinuation of the diet was reported in some individuals.

**Conclusions:**

Although there is no high-quality evidence of LC/KD efficacy in mood or anxiety disorders, several uncontrolled studies suggest possible beneficial effects. Robust studies are now needed to demonstrate efficacy, to identify clinical groups who may benefit and whether a ketogenic diet (beyond low carbohydrate) is required and to characterise adverse effects and the risk of relapse after diet discontinuation.

Mood and anxiety disorders are a major global health burden with a significant unmet need.^1^ There is an urgent requirement for more effective treatments.^2^ Current treatments include antidepressants, which have variable response rates^3^ and increasing concerns regarding severe withdrawal experiences,^4^ and psychological therapies, whose apparent efficacy might be affected by positive publication bias.^5^ In recent years, both preclinical and clinical evidence has emerged that supports the role of diet as an adjunctive therapeutic approach for mood disorders.^6^ Therapeutic options include versions of the Mediterranean diet used in the PREDIMED,^7^ SMILES^8^ and HELFIMED trials^9^ and two other Australian trials in young adults.^10,11^ Dietary approaches (whether alone or as augmentation) may be especially appealing to people with mood disorders who have mild to moderate symptoms and physical comorbidities, and therefore the potential impact on the wider population and economic factors may be significant.

One proposed dietary approach is carbohydrate reduction (typically with moderate increases in fat and protein), in the form of low carbohydrate (<130 g carbohydrate/day) and very low carbohydrate (<25–50 g/day) ketogenic diets. These have been defined and operationalised (Table 1). Low carbohydrate and ketogenic diets (LC/KD) dating back to the 1860s^12^ and the 1970s Atkins diet^13^ have shown efficacy in type 2 diabetes, obesity and metabolic syndrome. There is increasing interest, albeit with debate, especially regarding longer-term efficacy and potential adverse effects (such as hypercholesterolaemia, nutritional deficiencies and renal stones^14,15^). Interestingly, improvements in mood, energy, sleep, mental clarity and affect stability have been reported with use of LC/KD in type 2 diabetes and obesity.^16^

**Table 1:** Operational definitions: low carbohydrate and ketogenic diets (LC/KD)

Term	Abbreviation	Carbohydrate content, g/day and/or % daily intake of a nominal 2000 kcal diet	Comment
Moderate carbohydrate diet		300–400 g or 26–45%	Western-pattern diets typically contain over 300 g carbohydrate/day
Low carbohydrate diet	LC, LCD	<130 g or <26%	
Very low carbohydrate diet	VLCD	<30 g (some define 25–50 g) or <10%	Leads to nutritional ketosis if maintained beyond 4–7 days
Ketogenic diet	KD	Fat:carbohydrate:protein ratio calculated on an individual basis for children with epilepsy	High fat and very low carbohydrate: a ketogenic diet usually refers to the strict nutritional intervention for medication-resistant paediatric epilepsy^17,18^ (although the term is used more widely now). First described in 1921.^19^ Paediatric application requires specialist neurological and dietetic monitoring. Protein restricted: gluconeogenesis reduces ketosis. Diet modified according to child's weight and growth. Response rates are significant: at 3 months 55% are seizure-free and 85% have reduced seizures.^17^ Two types of ketogenic diet:^20^ ‘traditional’ ketogenic diet: strictly ultra-low carbohydrate, minimal protein intake, sufficient for growth; intense monitoring, vitamin, and mineral supplementation: classic ketogenic diet‘3:1’ and ‘4:1’ diets (ratio of fat to carbohydrate + protein) medium-chain triglycerides (MCTs) (1970s): better absorption and ketone yield, allowing less fat, more carbohydrate and protein Modified ketogenic diet: less restrictive, better adherence, equal efficacy: modified Atkins diet (MAD) 2003:^21^ carbohydrate <10 g/day child, <20 g/day adult, excluding fibre; protein allowed freely low glycaemic index treatment (LGIT) 2002:^22^ carbohydrate 40–60 g/day, restricted to foods with a glycaemic index <50, including fibre; normal amount of protein
Keto-adaptation			After Phinney:^23^ metabolic switch from carbohydrate to ketones as primary fuel source, stabilising over several weeks
Ketones			β-hydroxybutyrate (β-HB), acetoacetate, acetone
Ketosis			Presence of circulating ketones β-HB 0.5–3.0 mmol/l; physiological response to starvation or carbohydrate reduction (‘nutritional ketosis’)
Ketoacidosis			Distinct from nutritional ketosis: ketoacidosis defined as β-HB >20 mmol/l in the context of acutely destabilised type 1 diabetes, with severe hyperglycaemia and acidosis
Low carbohydrate high fat diet	LCHF		May be ketogenic (carbohydrates <25–50 g/day) or non-ketogenic (130–50 g/day); similar to the modified Atkins diet (MAD)^21^
Low carbohydrate ketogenic diet	LCKD, KLCHF		The terms LCHF, KLCHF, LC/KD and ketogenic diet have some overlap and may appear interchangeably in the literature

Source: modified from Accurso et al.^32^

The ketogenic diet has shown some efficacy for medication-resistant paediatric epilepsy, first suggested in 1921.^19^ An updated Cochrane review^17^ reported promising results for this intervention, with greater seizure reduction in those on a ketogenic diet compared with usual care: seizure freedom in up to 55% of children and seizure reduction in up to 85% after 3 months. However, conclusions were limited by the small number of studies and sample sizes and low to very low overall quality of evidence. Nevertheless, ketogenic diets are increasingly used for medication-resistant epilepsy, and there has been an increase in specialist ketogenic diet services.^24^ Together, research from epilepsy as well as studies describing improvements in mood and cognition in type 2 diabetes and obesity has stimulated interest in ketogenic diets for various psychiatric and neurological disorders, including cerebral glioma, Parkinson's disease, multiple sclerosis, chronic fatigue^25,26^ and mood disorders.^26–28^

Poor nutrition is a substantial driver of the global increase in non-communicable disorders.^1,29–31^ The pro-inflammatory and high carbohydrate content (typically >300 g/day^32^) of Western-pattern diets, especially high refined carbohydrates and ultra-processed foods, may be key factors.^31^

Importantly, it should be noted that the term ‘carbohydrates’ is now colloquially used to refer to highly refined carbohydrates, which make up a substantial part of Western diets. However, foods that are known to impart substantial health benefits due to their fibre content^33^ as well as dietary components used by gut bacteria to form molecules essential to health, such as polyphenols and resistant starch, including starchy vegetables, different whole-grain cereals and legumes, are also high in carbohydrates. These foods form a substantial part of Mediterranean diets, which have been linked to positive health outcomes.^34^ References to ‘carbohydrates’ throughout this review should be read with this important distinction in mind.

LC/KD are plausible therapies for mood disorders for several reasons. The relationship between mood disorders and carbohydrate intake is complex, with both acute and habitual effects at play. At a population level, sugar intake correlates with depression rates.^35^ High glycaemic index (GI) diets appear to negatively affect mood.^36–45^ Furthermore, ‘comfort eating’ is common in mood disorders and carbohydrate cravings are often present in seasonal affective disorder^46^ and atypical depression, which is common in bipolar disorder.^47^ Reducing carbohydrate intake might therefore alleviate mood disorder symptoms in some individuals. Indeed, animal models of depression suggest that a ketogenic diet might exert an antidepressant or anxiolytic effect.^48^ Case studies have also reported amelioration of psychotic symptoms following initiation of a ketogenic diet.^49^ However, randomised controlled trials (RCTs) are required to establish efficacy in reducing depression, anxiety and psychotic symptoms in psychiatric populations.

Although the efficacy of LC/KD for mood and anxiety disorders has not yet been established, public interest is increasing and clinicians may already encounter individuals utilising such approaches.^50^ A self-report uncontrolled survey of low carbohydrate diets in 1580 patients with a variety of conditions, including obesity and type 2 diabetes, reported significant improvements in mood, anxiety and energy and a reduction in antidepressant use (although psychiatric diagnoses were not provided).^16^ In contrast, a systematic review of LC/KD RCTs in obese and overweight adults without epilepsy or mood disorders (eight studies, *n* = 532, duration 8 weeks to 1 year) reported no overall psychological benefits.^51^ Two RCTs have also examined mood and behavioural effects of a ketogenic diet in paediatric epilepsy: Ijff et al reported reductions in anxious and mood-disturbed behaviour in those on a ketogenic diet,^52^ whereas Lambrechts et al found a tendency towards an increase in mood problems.^53^

Given this background, the aim of the current study was to systematically review low carbohydrate and ketogenic interventions in adults with mood and anxiety disorders to direct further avenues for research and to highlight uncertainties for clinical practice.

## Method

This review was conducted in accordance with the Preferred Reporting Items for Systematic Reviews and Meta-Analyses (PRISMA) guidelines^54^ and registered with the International Prospective Register of Systematic Reviews (PROSPERO: CRD42019116367). The following adjustments to the methods set out in the protocol were made during the review: (a) English language articles only were considered; (b) studies did not need to include a non-exposed comparison group to be eligible for inclusion, to widen the scope and generalisability of the review; (c) an adapted version of the Newcastle–Ottawa Scale was used for the risk of bias assessment,^55^ as it was more appropriate for the included study designs; and (d) grey literature was excluded in order to improve the quality of included studies.

### Eligibility criteria

Eligibility criteria: (a) primary research article; (b) published between database inception to 6 September 2022; (c) published in a peer-reviewed journal; (d) human adult participants (>18 years of age); (e) published in the English language; (f) individuals with mood or anxiety disorders, defined here as depression, anxiety, bipolar disorder and schizoaffective disorder; (g) low carbohydrate diet or ketogenic diet as the intervention. Exclusion criteria were papers in which the population had a primary neurodevelopmental disorder diagnosis (such as autism spectrum disorder or attention-deficit hyperactivity disorder) and animal studies.

### Information sources and search strategy

The search strategy was developed based on the research question ‘Do low carbohydrate or ketogenic diets confer mood benefits in mood and anxiety disorders?’ and the following PICOS (participants, interventions, comparisons, outcomes, study design) criteria:

- participants: adults with depression, anxiety, bipolar disorder or schizoaffective disorder
- interventions: low carbohydrate or ketogenic diets
- comparisons: if reported, ‘normal’ or ‘moderate’ carbohydrate diet, or Western-pattern diet, or placebo, or usual care, or befriending, or psychological support
- outcomes: reports of response, remission, relapse of symptoms (where reported, as defined by validated measures); adverse events; attrition
- study design: RCTs; if RCTs were not available, cohort studies, case series, case reports.

A systematic search of MEDLINE, Embase, PsycINFO and Cochrane was performed on 6 September 2022. No lower date search limit was set. The search strategy is detailed in Supplementary file 1, available at https://dx.doi.org/10.1192/bjo.2023.36.

### Study selection

Two reviewers (D.M.D. and M.H. and subsequently D.M.D. and J.K.-G.) independently assessed all retrieved records for inclusion using Covidence systematic review software (Veritas Health Innovation, Melbourne, Australia; www.covidence.org). First, reviewers screened titles and abstracts for eligibility. Any potentially eligible or ambiguous records were retained for the second round of screening, in which full texts were examined. Any discrepancies in the final articles to be included were discussed in a consensus meeting.

### Data extraction

Two authors (of D.M.D., J.K.-G., M.H. and W.M.) extracted the following information from included studies using Covidence software: author/date, study design, sample size, population characteristics (age, gender, comorbidities), type and duration of dietary intervention, adverse events and mental health-related outcomes, including measures of depression, anxiety and mood, and psychosis rating scales. The primary outcome was a clinically significant change in symptoms and/or change from baseline to last observation for relevant rating scales.

### Study risk of bias assessment

An adapted version of the Newcastle–Ottawa Scale was used for the risk of bias assessment,^55^ using items appropriate for cross-sectional, observational, cohort and non-randomised interventional study designs.

## Results

### Characteristics of included studies

**Fig. 1:**
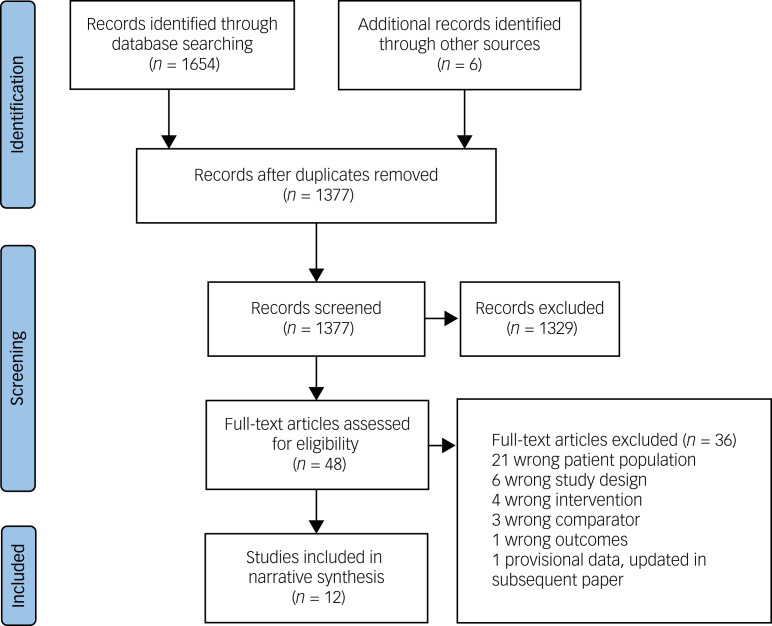
PRISMA flow diagram.

The results of the search strategy are summarised in Fig. 1. The search yielded 1377 articles, of which 48 full-text articles were assessed for eligibility. Of these studies, 36 were excluded. The 12 eligible studies are summarised in Table 2, stratified by study design. There were no RCTs. The studies comprised nine case reports, two cohort studies and one observational study. Bipolar disorder was the most studied mood disorder (*n* = 6 studies). All studies stated that the intervention was a ketogenic diet. However, there is no widely accepted definition of a ketogenic diet, carbohydrate intake was not reported in five studies and confirmation of ketosis was incomplete (blood ketone testing in two studies, urinary ketone testing in five, no confirmation in five). It is therefore possible that some of the interventions may have been a low carbohydrate rather than a ketogenic diet.

**Table 2:** Summary of included studies, stratified by study design

Study	Design	Participants	Dietary intervention and duration	Adherence and, where available, confirmation by measurement and presence of ketosis	Main mood and psychiatric symptom outcomes	Adverse effects
*Observational studies*						
Campbell & Campbell (2019)^50^	Online observational analytic study	*n* = 274; bipolar disorder (age and gender n.r.)	Ketogenic diet (85 participants, 165 online posts) compared with omega-3 or vegetarian diet (94 posts) Carbohydrate intake n.r. Duration not consistently reported, but where available (or derived) varied: 1–5 months, >6 months and >12 months	92.9% of participants ‘very likely’ to have achieved a state of ketosis	Remission or significant mood stabilisation reported in 56.4% of posts on ketogenic diet and 14.9% of posts on vegetarian diet or omega-3 supplementation In ketogenic diet, many detailed reports of the improvements experienced and several lasting for extended periods (months to years) In ketogenic diet, fewer episodes of depression (41.2%), improved clarity of thought and speech (28.2%), increased energy (25.9%) and weight loss in 25.9% Any mood destabilisation reported in 4.8% of posts on ketogenic diet and 10.6% of posts on vegetarian diet or omega-3 supplementation	No instances of hospital admission or care-seeking reported in posts for either diet group Difficulties in keto-adaptation reported in 10.6% of posts
*Cohort studies*						
Danan et al (2022)^59^	Retrospective cohort study	*n* = 31; psychiatric in-patients: 13 bipolar II disorder, 12 schizoaffective disorder, 7 MDD (mean age: 50 years; 71% female)	Ketogenic diet (carbohydrate <20 g/day) Duration: at least 14 days, mean 59.1 days	3 participants discontinued before 14 days. Ketosis (measured via urinary ketones) achieved by 64% of remaining participants. Dietary adherence characterised as excellent (39%), good (43%) or fair (18%)	Symptoms of depression and psychosis and overall clinical severity significantly reduced All patients who completed the HRSD achieved a reduction of ≥4 points and 95% achieved a reduction of ≥6 points. All patients who were assessed with the MADRS achieved a reduction of ≥6 points Number or dose of psychotropic medications was reduced in 64% of participants	Most patients reported initial symptoms of keto-adaptation (headache, insomnia, irritability, excitation, dizziness, carbohydrate cravings), which resolved within 2 weeks; beyond this, 13% reported adverse effects (fat intolerance, diarrhoea, vomiting)
Kunin (1976)^68^	Cohort study	*n* = 73; psychiatric out-patients with symptoms of anxiety, depression and ‘dysperception’ (age and gender n.r.)	Ketogenic diet (carbohydrate 0 g/day) until ketosis reached (or maximum 5 days); then ‘optimal carbohydrate level’/low carbohydrate (<120 g/day, mean 52 g/day), duration n.r.; then higher carbohydrate diet (>120 g/day), duration n.r.	2 participants discontinued within 5 days. Ketosis measured via urinary ketones	Symptoms of anxiety, depression and dysperception improved with ketogenic and ‘optimal carbohydrate level’ diets in 82% of participants	60% of participants reported transient adverse effects, including fatigue, nausea, weakness, headache and palpitations; improved after administration of potassium salts
*Case reports*						
Chmiel (2021)^64^	Case report	*n* = 1; ultra-rapid cycling bipolar disorder (not further specified but suggestive of bipolar II disorder) (27-year-old male)	Low carbohydrate high fat diet for 1 year; then ketogenic diet (carbohydrate <30 g/day, 2500 kcal, 15% protein, 80% fat, 5% carbohydrate) for 1 year; then ketogenic diet with 1 day fast every 7–10 days for 1 year	Average reported blood ketones: year 1 β-HB = 0.3–0.5 mmol/L; year 2 β-HB = 1.5–3 mmol/L; year 3 β-HB = 5 mmol/L on fasting days	Depression reduced, mood stabilised, increased energy, improved sleep, cognitive function and concentration, and elimination of anxiety; no hypomania Remission of depression in year 3. Discontinuation of quetiapine (previously up to 300 mg/day) and reduction of lamotrigine to 100 mg/day (previously up to 300 mg/day)	n.r.
Cox et al (2019)^65^	Case report	*n* = 1; type 2 diabetes and comorbid MDD (65-year-old female)	Ketogenic diet (65% fat, 25% protein, 10% carbohydrate) for 3 months	Blood ketones averaged 1.5 mmol/L by week 12	PHQ-9 score decreased from 17 (baseline) to 0 (week 12); reported increased self-efficacy and self-confidence, increased energy, improved sleep, stability in mood and clearer cognition	n.r.
Ehrenreich (2006)^60^	Case report	*n* = 1; panic disorder (47-year-old female)	Atkins diet (carbohydrate intake n.r.) for 4 weeks	Not assessed, but 17 lb (7.7 kg) weight loss reported	Increase in baseline anxiety level over the course of the diet	Internal sensation of ‘shakiness’, frequent panic attacks; resolved on cessation of diet
Kraft & Westman (2009)^66^	Case report	*n* = 1; schizophrenia and comorbid depression (70-year-old female)	Ketogenic diet (carbohydrate <20 g/day) for 12 months	Reported 2–3 isolated episodes of non-adherence lasting several days; 10 kg weight loss reported	Reported increased energy and no longer experienced auditory or visual hallucinations	n.r.
Palmer (2017)^58^	Case report	*n* = 2; schizoaffective disorder (33-year-old male, 31-year-old female), both with comorbid MDD	Ketogenic diet (carbohydrate intake n.r.); male for 12 months; female for 4 months	Male discontinued diet on 5 occasions and experienced relapse of positive and negative symptoms within 1–2 days; female discontinued diet once and developed paranoia and delusions	Reductions in hallucinations and delusions, improved mood, energy and concentration. PANSS scores reduced from 98 to 49 in the male and from 107 to 70 in the female	n.r.
Phelps et al (2012)^63^	Case report	*n* = 2; bipolar II disorder (69-year-old female, 30-year-old female)	Ketogenic diet (70% fat, 22% protein, 8% carbohydrate for 30-year-old; details n.r. for 69-year-old); 69-year-old for 2 years; 30-year-old for 3 years	Ketosis measured via urinary ketones: ranged between 0 and 80 mg/dL (over course of 7 months in 69-year-old; ketosis measurement n.r. for 30-year-old)	Sustained mood stability; diet enabled both to discontinue lamotrigine; no increase in anxiety.	None
Pieklik et al (2021)^67^	Case report	*n* = 1; ‘mood disorder’ (not specified) with comorbid emotion dysregulation, body dysmorphic disorder and an eating disorder (21-year-old female)	Very low calorie ketogenic diet (details n.r.) (carbohydrate intake n.r.) for 4 weeks	Did not fully comply with diet (details n.r.); no measures of ketosis; weight reduced from 113.5 kg to 102 kg, some metabolic parameters improved	Partial improvement in well-being, mood stabilised, decreased anxiety, no suicidal thoughts. BDI score improved from 40 (severe depression) to 23 (moderate) Other interventions included sertraline, trazodone, metformin (timing, doses and duration n.r.), psychotherapy and psychoeducation	Did not experience adverse effects but did not want to continue nutritional intervention; overall ‘therapeutic cooperation’ reported as ‘difficult’
Saraga et al (2020)^62^	Case report	*n* = 1; bipolar I disorder (60-year-old female)	‘Mildly ketogenic’ diet (ratio of grams of fat to grams carbohydrate + protein of 2–3:1); duration n.r.	Ketosis measured via urinary ketones: 0.05–0.4 g/L	Decreased anxiety, maintenance of euthymia; enabled discontinuation of Sertindole; patient describesd clear improvement on both depressive and manic symptoms	n.r.
Yaroslavsky et al (2002)^61^	Case report	*n* = 1; in-patient with bipolar I disorder (49-year-old female), treatment-resistant, rapid cycling	Ketogenic diet (carbohydrate intake n.r.) for 4 weeks	Urinary ketosis was not confirmed, nor was there any weight loss. Patient adherence reported as very good	No clinical improvement	n.r.

β-HB, beta-hydroxybutyrate; BDI, Beck Depression Inventory; GAD-7, seven-item Generalised Anxiety Disorder questionnaire; HRSD, Hamilton Rating Scale for Depression; MADRS, Montgomery–Åsberg Depression Rating Scale; MDD, major depressive disorder; n.r., not reported; PANSS, Positive and Negative Syndrome Scale; PHQ-9, nine-item Patient Health Questionnaire (depression module).

### Risk of bias assessment

Cohort and observational studies were assessed for risk of bias (Supplementary file 2). Owing to the heterogeneity in study designs, we did not provide a quality score for each study and instead provided a qualitative assessment.^56^ None of the studies employed representative sampling techniques and confounders were not controlled for. Changes in anxiety, depression and/or psychotic symptoms were measured via validated questionnaires in four studies.^57–59^ One study additionally measured depression symptoms and illness severity via clinical interview.^59^

### Synthesis of results

Considering the methodological heterogeneity between studies, a quantitative appraisal was not possible. Findings were therefore synthesised narratively, clustered by study type and then stratified as either ‘no efficacy’ or ‘possible efficacy’ of the intervention. Discontinuation effects and adverse events were described where reported (for clarity, these have been grouped together, given the limited data).

#### Case reports

##### No efficacy of LC/KD intervention

Two case reports reported no apparent benefits of the intervention (duration 4 weeks each). One report described a participant with panic disorder (*n* = 1)^60^ on a ketogenic diet who experienced negative effects (internal ‘shakiness’, increase in anxiety and recurrence of panic attacks, despite increasing sertraline dose). These symptoms resolved on discontinuation of the ketogenic diet. The other case report described an unsuccessful attempt at a ketogenic diet in a hospital in-patient with rapid cycling bipolar disorder (mania predominant) (*n* = 1).^61^ Ketosis was not achieved despite reported adherence to the diet, so this may have been a low carbohydrate diet rather than a ketogenic one.

**Table 3:** Uncertainties regarding the use of LC/KD for mood and anxiety disorders in clinical practice

**Efficacy**
Which clinical groups may (or may not) benefit? Can/should clinicians recommend LC/KD as ‘off-label’ therapy, and if so when and when not? What are the clinical and medicolegal implications? How should clinicians respond to patients preferring LC/KD over other therapies? (potential risk if those with severe symptoms choose LC/KD in place of evidence-based therapies). Do LC/KD have inherent antidepressant, anxiolytic and/or mood stabilising effects, or are these secondary to weight loss, improved energy (and behavioural activation)? ‘Dose’: is ketosis required or is a non-ketotic low carbohydrate diet effective? Times to response, response rates, numbers needed to treat and numbers needed to harm for LC/KD compared with other therapies. Could LC/KD augment/replace other therapies? Long-term efficacy and efficacy in relapse prevention. Efficacy in individuals without obesity/metabolic syndrome/type 2 diabetes. Could LC/KD improve cognitive problems in mood disorders?
**Adherence**
Strategies to aid LC/KD induction and maintenance (is this more challenging for individuals with mood disorders compared with those following LC/KD for obesity/type 2 diabetes but without a mood disorder?) Speed and severity of relapse on discontinuation (case reports suggest this may be rapid and significant). Managing mood instability if LC/KD adherence is intermittent.
**Adverse effects**
Impact and management of induction symptoms. Restricted diet might exacerbate disordered eating. Weight loss likely: beneficial in people with obesity/metabolic syndrome but problematic if normal or low baseline BMI (and is this a contraindication if so?). Gut health: constipation, altered gut microbiota and gut metabolites with negative effects. Cardiovascular risk is uncertain: beneficial effect on inflammatory markers but variable effect on lipids.
**Potential effects on pharmacotherapy**
A ketogenic diet may affect pharmacokinetics, for example lowering valproate levels, needing dose adjustment. Ketogenic diet-related diuresis may cause electrolyte changes, which could affect lithium levels and increase adverse effects. LC/KD often lowers blood pressure, weight and glucose levels – antihypertensive and diabetic medications will need to be adjusted or withdrawn.

##### Possible efficacy of LC/KD intervention

Seven case reports reported possible efficacy (duration of diet between 1 month and 3 years). Three described substantial improvements in symptoms in individuals with bipolar disorder (*n* = 4), including greater mood stability, reductions in frequency of mood episodes and decreased anxiety.^62–64^ Four patients discontinued or reduced their antipsychotic or mood stabiliser medication. Ketosis was measured by blood β-hydroxybutyrate in one report^64^ and by urinary ketones in two^62,63^ reports. The other four case reports described a female with type 2 diabetes and comorbid major depressive disorder,^65^ a female with schizophrenia and comorbid depression,^66^ a female with a mood disorder and comorbid emotion dysregulation, body dysmorphic disorder and an eating disorder,^67^ and two people (one male, one female) with schizoaffective disorder and comorbid major depressive disorder.^58^ Reported benefits included improved mood, energy, concentration and cognition, as well as reduced psychotic symptoms in those with schizoaffective disorder.

#### Cohort studies

##### No efficacy of LC/KD intervention

No studies.

##### Possible efficacy of LC/KD intervention

Possible efficacy was reported in a cohort study of ketogenic and low carbohydrate diets in out-patients with anxiety, depression and ‘dysperception’ (*n* = 73; duration not reported)^68^ and in a study of a ketogenic diet in in-patients with bipolar II disorder, major depressive disorder or schizoaffective disorder (*n* = 31, mean duration 59.1 days).^59^ The first study reported improvements in anxiety, depression and ‘dysperception’ in 82% of participants. Similarly, the second study reported improvements in self- and clinician-rated depression, as well as overall severity of illness in participants with mood disorders; further, around two-thirds of participants reduced the number or dose of psychotropic medications by the end of the intervention.

#### Observational studies

##### No efficacy of LC/KD intervention

No studies.

##### Possible efficacy of LC/KD intervention

In the only observational study in the review, Campbell & Campbell^50^ reported an analytic study of posts on online forums about a ketogenic diet versus omega-3 supplementation or a vegetarian diet from people with bipolar disorder (*n* = 274; reporting of diet duration incomplete but where available it was between 1 month and >12 months). Remission or significant mood stabilisation was reported in 56.4% of posts discussing a ketogenic diet, compared with 14.9% of posts on a vegetarian diet or omega-3 supplementation. Posts discussing mood destabilisation were few overall, but more commonly associated with a vegetarian diet or omega-3 supplementation than a ketogenic diet.

### Effects of discontinuation of LC/KD intervention

Of the ten studies that reported symptom improvement with LC/KD, only four reported information on symptom changes on stopping LC/KD. Campbell & Campbell^50^ reported recurrence of bipolar disorder symptoms in 7.1% of participants on stopping a ketogenic diet and Kunin^68^ reported recurrence of symptoms in 82% of participants with depression, anxiety and ‘dysperception’ on cessation of a low carbohydrate diet. Two case reports reported mixed results. Kraft & Westman^66^ reported no recurrence of psychotic symptoms during dietary relapses in a person with schizophrenia and comorbid depression; however, Palmer^58^ reported recurrence of psychotic symptoms during dietary relapse in two individuals with schizoaffective disorder, despite continuing antipsychotic medication in one of them. Symptoms resolved when ketosis was induced; however. an increase in the individual's antipsychotic medication dosage may also explain this improvement.

### Adverse events

Adverse events were not reported systematically (Table 2**)**. Six studies did not report on adverse events.^58,61,62,64–66^ Two studies reported no adverse events (*n* = 3).^63,67^ Four studies reported adverse events, including: transient symptoms associated with keto-adaptation (e.g. fatigue, nausea, headache, palpitations) (*n* not recorded),^50,59,68^ ‘shakiness’, increase in anxiety and recurrence of panic attacks (*n* = 1),^60^ fat intolerance (*n* = 2) and gastrointestinal symptoms (*n* = 2).^59^

## Discussion

This systematic review examined the efficacy of low carbohydrate and ketogenic diets (LC/KD) in individuals with mood and anxiety disorders. Despite anecdotal reports and biological plausibility, little research has been conducted to date and no high-grade evidence was found. Heterogeneity and data quality limit interpretation of the included studies, and statistical analysis including meta-analysis was not possible, leading to a narrative review.

It should be noted that nutritional intake and measurements of ketosis were variable and incomplete, and although the interventions were stated to be ketogenic diets, there was no consistent threshold for ketone levels to define ketosis and it is possible that some individuals were on low carbohydrate rather than ketogenic diets, either throughout the duration of the study or periodically, and did not reach the required threshold for ketosis.

Although two studies suggested no benefits of LC/KD, several case reports, cohort studies and the observational study suggest possible efficacy of a ketogenic diet in bipolar disorder, schizoaffective disorder, anxiety disorders and unipolar depression. Reported therapeutic effects of a ketogenic diet include mood stability, increased energy and concentration, and reductions in symptoms of anxiety, depression and psychosis. Relapse occurred in several individuals on discontinuation of the diet. Adverse effects were not systematically reported but included fatigue, nausea and headaches on induction of ketosis, although this was not universal.

Potential benefits of a ketogenic diet reported here concur with previous research exploring self-reported benefits of low carbohydrate diets in other populations.^16,69^ The present paper expands on reviews by Bostock et al^26^ and Brietzke et al^28^ by incorporating low carbohydrate interventions (not just a ketogenic diet) and a wider clinical context: this may increase generalisability to community samples of individuals with mood disorders. In contrast, in individuals without mood disorders, El Ghoch et al^51^ found no overall evidence of psychological benefits of LC/KD (eight studies, *n* = 532; duration 8 weeks to 1 year). However, in El Ghoch et al's review: (a) two studies did not meet criteria for a low carbohydrate diet,^70,71^ (b) ketosis was measured in only five studies^72–76^ and (c) several included studies utilised the 24 h Food Frequency Questionnaire, the validity of which has been questioned.^77^ Alternatively, it might be that LC/KD are efficacious only in individuals with clinical mood disorders and thus more severe psychopathology, but not in non-clinical samples with milder symptoms. It is also possible that LC/KD are more useful in those with greater metabolic burden, such as threshold metabolic syndrome.^78^

### Context and mechanisms

Any response to LC/KD may be modulated via multiple mechanistic pathways. For example, LC/KD have been suggested to reduce inflammation, which is increasingly acknowledged to be involved in the development of depression and, in particular, treatment-resistant depression.^79–82^ A systematic review^83^ showed that low glycaemic index and low glycaemic load diets were associated with reduced inflammatory markers in 5 out of 9 observational studies; 3 of 13 interventional trials showed significant anti-inflammatory effects and 4 suggested beneficial trends. Reduced inflammation correlates with reduced depression in obesity and overweight,^84,85^ which may be related to reduced inflammatory adipokines.^86,87^

Mitochondrial energy generation – which can be affected by ketosis – is also important, because mood disorders, especially bipolar disorder, are associated with abnormal mitochondrial energy generation.^88,89^ Depression is characterised by reduced energy generation, and mania is characterised by increased mitochondrial biogenesis.^90^ A ketogenic diet has the potential to increase the efficiency of mitochondrial biogenesis and may target this core pathway.^88,91,92^

Furthermore, improvements in insulin signalling conferred by LC/KD (as evident in treatment of obesity and type 2 diabetes) may be associated with mood benefits. Calkin et al showed that individuals with bipolar disorder and comorbid type 2 diabetes or insulin resistance had three times higher odds of chronicity and rapid cycling and more than eight times the odds of lithium resistance, even after controlling for antipsychotic use and body mass index (BMI).^93^ Subsequently, in a first RCT, Calkin et al showed that reversing insulin resistance by adding metformin in treatment-resistant bipolar depression had a large effect size on clinician-rated depressive symptoms – but only in participants whose insulin resistance was reversed.^94^ Reversing insulin resistance is clearly a plausible outcome of a low carbohydrate diet, not requiring ketogenic diet levels of carbohydrate reduction. In patients at risk, low carbohydrate diets can be advocated for prevention of obesity, metabolic syndrome and type 2 diabetes, with the possible additional benefit of an antidepressant effect in bipolar disorders. Thus, the role of insulin resistance in mood disorders may justify the application of LC/KD interventions in this population.

Ketosis has neuroprotective mechanisms in epilepsy,^95^ so it is possible that ketosis may also confer positive mood outcomes in non-epileptic disorders. As proof of concept, short-term fasting (generating ketosis) may improve mood and induce mild euphoria in individuals with mood disorders.^96^ Longer-term ketosis-related mood stabilisation in bipolar disorder is reflected in the self-reports analysed by Campbell & Campbell.^50^ Putative mechanisms of ketosis on mood may involve several factors, including increased fatty acid synthesis and oxidation, neurotransmitters, ion channels, mitochondrial genesis, cell signalling, second messengers and reduced oxidative stress.^97,98^ Notably, the seizure-reducing effects of ketosis in epilepsy have not been clarified. Ketosis also induces brain-derived neurotrophic factor (BDNF) gene expression;^99^ this is important as a low BDNF level adversely affects neuroplasticity^100^ and BDNF increases with effective treatment.^101^ Weight loss associated with a ketogenic diet might also be a relevant mechanism, although reports of significant mood improvement as early as 4 days into a ketogenic diet, which precedes any significant weight loss, is consistent with ketosis-mediated effects on mood rather than weight loss *per se*. A further factor potentially playing a role in mediating effects of a ketogenic diet on mood is reduced appetite,^102^ as discussed in more detail below.

### Limitations

There are several limitations to this review, partly related to the low number of studies on this topic and their general poor quality (for example, lack of non-exposed control groups and consideration of confounding factors). The broad inclusion criteria, including lower levels of evidence, may have resulted in heterogeneity between studies and variable data quality.

We pragmatically grouped depression, anxiety, bipolar disorders and schizoaffective disorders together, given some overlapping symptomatology, because of the paucity of LC/KD evidence for individual disorders. A case could also be made against the inclusion of studies with limited information on psychiatric diagnoses,^57,68^ as the results of these studies may not be generalisable to individuals with mood disorders without such comorbidities.^103^

The effects of LC/KD on mood disorders might also involve confounding factors, which were rarely assessed in the studies included in this review. These include concurrent pharmacotherapy, reduced alcohol intake (high carbohydrate content), reduced hunger (ketosis effect on appetite hormones),^103^ weight loss, improved sleep and (in those on a well-formulated low carbohydrate diet) presumed increased intake of folate, micronutrients, vitamin D and omega-3 fatty acids (LC/KD typically increase intake of leafy green vegetables and fish).

The exclusion of certain foods may also be an important confounder. For example, reduced gluten intake in LC/KD has been suggested to be a confounder in a limited number of studies, as there are reports of anti-gluten antibodies in psychosis^104^ and bipolar disorder.^105^ Furthermore, an unnecessary gluten-free diet (which LC/KD might entail) might increase the risk of nutritional deficiencies^106^ and gastrointestinal and cardiovascular side-effects.^107,108^

Another potential confounder is the potentially low content of fermentable oligosaccharides, disaccharides, monosaccharides and polyols (FODMAPs) in LC/KD (depending on the dietary formulation). FODMAPs, poorly absorbed short-chain carbohydrates (for example in bread), may cause bloating and fatigue in some individuals and are implicated in the symptomatology of irritable bowel syndrome (IBS): FODMAP reduction or exclusion improves IBS.^109^ The relevance here is that IBS and various psychiatric disorders (notably, anxiety disorders and, possibly, current major depression) are often comorbid.^110^ Interestingly, the omission of FODMAPs confounds gluten-free diet trials in IBS^109^ and although there is uncertainty regarding the psychiatric effects of FODMAPs,^111^ it could be argued that omitting FODMAPs might confound any apparent psychiatric effects of LC/KD.

Three of the included studies^58,61,64^ also report intake of exogenous ketogenic food supplements: medium-chain triglycerides. The role of exogenous ketogenic food supplements is unclear^112^ and it must be noted that these compounds are high in saturated fatty acids, which may increase the risk of cardiovascular disease.^113^

Finally, no studies assessed gut microbiota, which may affect mood and/or anxiety.^113^ A ketogenic diet may be associated with changes in alpha diversity and beneficial microbial metabolites such as short-chain fatty acids,^114^ although the data are limited and clinical implications uncertain. Further discussion is beyond the scope of this review.

### Implications for clinical practice

Clinicians in primary care, mental health services and dietetics/nutrition may increasingly encounter patients who wish to follow LC/KD for depression, anxiety, bipolar disorder or schizoaffective disorder. Shared decision-making and good communication of the current absence of data able to clearly support or refute the efficacy of a ketogenic diet is important. Some of these issues are discussed below and in Table 3.

**Fig. 2:**
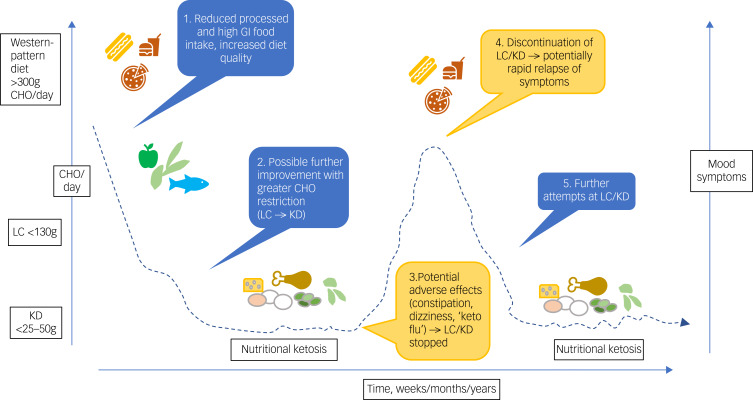
Schematic illustration of potential impact of low carbohydrate and ketogenic diets (LC/KD) on mood symptoms. Symptoms may improve as carbohydrate (CHO) intake drops, for example when intake is <25–50 g/day for >4 days, inducing nutritional ketosis, although further evidence is required. Adverse effects may be challenging for some, causing discontinuation of the diet and relapse of mood symptoms. Some individuals may make further attempts at a low carbohydrate (LC) or ketogenic diet (KD). Clinical supervision is required to manage potential adverse effects and adherence problems across all phases of ketogenic dietary interventions.

Low carbohydrate diets – and especially ketogenic diets – are by nature highly restricted diets, whereas ‘whole of diet’ approaches are preferable and more likely to be sustainable in people with mood disorders.^115^ As illustrated in Fig. 2, adherence to these diets, especially ketogenic diets, may be challenging, leading to either premature cessation (before potential benefits) or after therapeutic nutritional ketosis has been reached (which could equally apply to other treatment modalities).

Adverse effects include (a) early ‘flu-like’ symptoms during induction of ketosis,^112,116^ predominantly due to early electrolyte changes (especially sodium diuresis), contributing to fatigue, constipation, cramps, palpitations and postural hypotension, and (b) perhaps renal stones, gout, osteoporosis and cardiovascular and gastrointestinal disease (see below) in the longer term.

The data on longer-term LC/KD adherence in various clinical populations remain uncertain. In epilepsy, a Cochrane review (*n* = 932 children and adults) found that adults following a ketogenic diet may be up to five times more likely to drop out of studies compared with those receiving usual care.^17^ In contrast, an online nutritional ketosis programme for adults with type 2 diabetes (*n* = 349) reported 74% adherence at 2 years (*n* = 194/262), compared with 78% adherence for those in usual care (*n* = 68/87).^117^ The level of support provided to help participants follow the dietary intervention is likely to affect attrition rates, but this factor has not been systematically studied in relation to ketogenic diets. Further, these data have uncertain generalisability to patients with mood disorders, as it is possible that LC/KD adherence may be more challenging for individuals with such disorders. Pertinently, carbohydrate cravings and impulsivity present in many psychiatric disorders^118^ would presumably increase the chance of consumption of high carbohydrate foods, with resultant dietary relapse (as shown in Fig. 2). Furthermore, repeated attempts at LC/KD with initial efficacy but relapse after challenges might exacerbate feelings of guilt and failure. It would therefore be crucial to develop strategies to overcome these challenges in clinical populations.

Several practical aspects, especially of ketogenic diets, warrant consideration. First, daily carbohydrate counting (which may be required to help maintain ketosis) might exacerbate disordered eating and/or obsessive traits. Second, although confirmation of ketosis might be preferable, especially for individuals new to ketogenic diets, this might unnecessarily ‘medicalise’ normal eating patterns.

In addition, measurements of ketosis are temporally variable, may be imprecise (especially in mild nutritional ketosis, in contrast to diabetic ketoacidosis) and may pose logistical challenges. Methods for confirming ketosis include urine (acetoacetate), blood (β-hydroxybutyrate) and breath (acetone) analyses. Urinary ketone testing strips, although popular (they are low cost, non-invasive and easy to use), are unreliable: in mild dietary ketosis sensitivity is 35–76% and specificity 78–100%.^119^ Blood ketone monitoring (venous, capillary) is more reliable: depending on the magnitude of ketosis, sensitivity is 98–100% and specificity 85–93.3%;^120^ however, it may be impractical in the real world because of the cost and discomfort of blood tests or finger-prick testing (for related reasons, in diabetes, regular finger-prick glucose testing is being replaced by indwelling interstitial glucose devices,^121^ but interstitial ketone monitoring devices are not currently available). Breath ketone analysis is a relatively recent technology with uncertain accuracy and some devices are not registered with the Food and Drug Administration.^122^

The involvement of dietitians was an important part of clinical trials of dietary interventions in participants with depression such as the SMILES and HELFIMED trials.^8,9^ This is also supported by a recent meta-analysis that reported that dietitian-led intervention trials showed a greater improvement in depressive symptoms.^79^ This may be resource-intensive in naturalistic settings, but group sessions or internet-based delivery may be acceptable alternatives.^123^

Importantly, a paucity of longitudinal studies has led to uncertainty and debate regarding aspects of long-term LC/KD safety, especially potential cardiovascular disease (CVD) and gastrointestinal risks from increased saturated fatty acid intake and reduced dietary fibre. Increased saturated fatty acid intake has an unpredictable effect on lipids (a surrogate marker for CVD).^124–128^ Reduced dietary fibre has potentially negative effects on gut metabolites and microbiota, which may increase the risk of CVD, gastrointestinal and other diseases.^129–131^

Finally, the potential effect of LC/KD on medications is important. Careful monitoring is essential and medication amendments may be warranted. For example, a ketogenic diet may affect pharmacokinetics and valproate levels.^132,133^ Ketogenic diet-related diuresis (especially during keto-adaptation) may cause electrolyte changes that could affect lithium levels. These factors might pose additional challenges if individuals have intermittent adherence to LC/KD.

### Recommendations for further research

Robust RCTs are required to investigate the effects of low carbohydrate and ketogenic dietary interventions (with cross-over) on short- and longer-term outcomes in dysthymia, generalised anxiety disorder, major depressive disorder, atypical depression, bipolar disorder and schizoaffective disorder. Rating scale subgroup analysis might identify specific LC/KD effects on mood, anxiety, energy and sleep. The comparison of low carbohydrate diets with ketogenic diets is especially pertinent because, if efficacious, the less restrictive nature of low carbohydrate diets could increase generalisability. It would also be important to determine whether comorbid obesity/metabolic syndrome affects efficacy, adherence and relapse rates. Continuous interstitial glucose monitoring, such as in the large (*n* = 1100) multinational PREDICT I nutrition study,^134^ combined with daily self-rated mood measures (for example, the True Colours app^135^) might enable a fine degree of personalisation. This might, for example, show that individuals with impaired carbohydrate metabolism (a larger post-prandial glucose incremental area under the curve^136^) are more likely to respond to LC/KD, but (perhaps) not those with normal response.

These studies may be informed by the first RCT of a ketogenic diet in psychosis (NCT03873922), an open label trial of a ketogenic diet in euthymic bipolar disorder and schizophrenia (NCT03935854) and a feasibility study of a ketogenic diet in bipolar disorder (ISRCTN61613198). LC/KD effects on pharmacotherapy also require further study.

Neuroimaging may be informative. In people with epilepsy treated with a ketogenic diet, magnetic resonance spectroscopy (MRS) shows an increase in cerebral energy,^137,138^ which is thought to be a potential mediator of ketogenic diet efficacy. MRS might therefore also be relevant to ketogenic diets in populations with mood disorders, especially bipolar disorder, given the anomalies of mitochondrial biogenesis.^88,91,92^ Positron emission tomography or functional magnetic resonance imaging might also inform any ketosis-mediated neuroplasticity and neuroprotection, including changes in higher cortical function and cognition.^139^ Imaging research could also target the putative anti-inflammatory actions of LC/KD, focusing on changes in microglial activation^85,140,141^ with translocator protein density^142^ in the dorsal anterior cingulate cortex and prefrontal cortex (PFC). Functional BDNF imaging (amygdala, hippocampus, PFC) would add further information on potential mechanisms of LC/KD.^143,144^ Measurement of lactate might be the simplest biomarker of bioenergetics.^145^

Further research on LC/KD in mood disorders should include appetite hormones (including insulin, leptin and ghrelin), because of the interplay between LC/KD, appetite, insulin sensitivity and mood disorders.^87,146,147^ These factors might also link with inflammation noted above. Briefly, the role of appetite hormones and mood disorders is complex and may differ between disorders.^147^ For example, Cordas et al showed that reduced leptin is present in depression, especially atypical and bipolar depression; additionally, leptin is associated with current depression.^148^ Conversely, Pasco et al showed that leptin is elevated in women with a history of unipolar major depression and elevated leptin predicts future depressive disorder.^149^

Notwithstanding this, leptin has multiple roles in glucose homeostasis, including improving insulin sensitivity,^146^ which, as noted above, has been shown to be important in treating bipolar depression.^93,94^ LC/KD reduces leptin while also improving sensitivity to leptin.^150^ The role of ghrelin in mood disorders is unclear,^147^ but ketosis rapidly suppresses ghrelin, reducing appetite within a few days.^102^

Thus, the degree and rate of change of appetite hormones and mood disorder symptoms in various disorders during LC/KD interventions would be instructive. These findings may have important translation potential (Table 3) and might lead to formulation of dietary recommendations for mood disorders (as suggested for those with chronic pain).^151^

Finally, the effect of LC/KD on overall healthcare costs in mood disorders would also be of interest, as these costs have been shown to be reduced by LC/KD in people with type 2 diabetes.^152^

This review was conducted in the context of debate around many aspects of nutrition, especially ‘low carb’ diets, and mounting scientific and public interest. There is a very limited and low-quality evidence base on the efficacy of KD in mood disorders. Efficacy and adherence are unclear, and there are concerns about potential adverse effects. Nevertheless, some individuals already employ these strategies, and definitive and rigorous trials are needed to clarify safety and efficacy to guide clinical practice.

## Data Availability

Data availability is not applicable to this article as no new data were created or analysed in this study.

## Supplementary material

For supplementary material accompanying this paper visit http://doi.org/10.1192/bjo.2023.36. click here to view supplementary material
