# Supplementary material for: Efficacy of low carbohydrate and ketogenic diets in treating mood and anxiety disorders: systematic review and implications for clinical practice
Source: BJPsych Open. 2023 Apr 17;9(3):e70. doi: 10.1192/bjo.2023.36 (PMC10134254; doi:10.1192/bjo.2023.36)
Supplement: Supplementary file 1 [file bjosup.zip › S2056472423000364sup001.docx]

**Supplementary file 1**

**Search strategy** Search date 06.9.2022

**1. Database: Ovid MEDLINE(R), Embase, PsycINFO**

1. affective symptoms/ or aggression/ or depression/ or mental fatigue/ or obsessive behavior/
2. exp depression/
3. "depression (emotion)"/ or exp major depression/ or sadness/ or exp separation reactions/
4. (depression or depressed or depressive).mp. [mp=ti, ab, ot, nm, hw, kf, px, rx, ui, an, tn, dm, mf, dv, kw, tc, id, tm]
5. exp Anxiety/
6. exp anxiety/
7. exp anxiety disorder/
8. exp anxiety disorders/ or exp anxiety/ or anxiety management/
9. 9  (anxiety or anxious).mp. [mp=ti, ab, ot, nm, hw, kf, px, rx, ui, an, tn, dm, mf, dv, kw, tc, id, tm]

10  dysthymia/

11  dysthymic disorder/

12  dysthymia.mp.

13  dysthymic disorder.mp.

14  low mood.mp.

15  mental fatigue.mp.

16  exp Mood Disorders/

17  exp affective disorders/

18  mood disorder$.mp.

19  affective disorder$.mp.

20  or/1-19

21  Ketogenic Diet/

22  Ketogenic Diet.mp.

23  Diet, Paleolithic/

24  paleolithic diet/

25  paleolithic diet.mp.

26  palaeolithic diet.mp.

27  paleo diet.mp.

28  caveman diet.mp.

29  (stone-age diet or stone age diet).mp. [mp=ti, ab, ot, nm, hw, kf, px, rx, ui, an, tn, dm, mf, dv, kw, tc, id, tm]

30  Diet, Carbohydrate-Restricted/

31  low carbohydrate diet/

32  carbohydrate-restricted diet.mp.

33  carbohydrate restricted diet.mp.

34  low carbohydrate diet.mp.

35  low carbohydrate high fat diet$.mp.

36  high fat diet.mp.

37  lchf diet.mp.

38  vlchf diet.mp.

39  lckd diet.mp.

40  hflc diet.mp.

41  high fat low carbohydrate diet.mp.

42  or/21-41

43  20 and 42

44  limit 43 to humans [Limit not valid in PsycINFO; records were retained]

45  limit 44 to english language

46  remove duplicates from 45

47  (mice or mouse or rat or animal).mp. [mp=ti, ab, ot, nm, hw, kf, px, rx, ui, an, tn, dm, mf, dv, kw, tc, id, tm]

48 46 not 47

**2. Search Name: Ketogenic diet and depression or anxiety Description: Cochrane CENTRAL**

ID Search Hits

#1  MeSH descriptor: [Affective Symptoms] this term only

#2  MeSH descriptor: [Aggression] this term only

#3  MeSH descriptor: [Mental Fatigue] this term only

#4  depression or depressed or depressive

#5  MeSH descriptor: [Anxiety] explode all trees

#6  MeSH descriptor: [Anxiety Disorders] explode all trees

#7  anxiety or anxious

#8  MeSH descriptor: [Dysthymic Disorder] this term only

#9  dysthymia

#10  "dysthymi* disorder*"

#11  "low mood"

#12  MeSH descriptor: [Mood Disorders] explode all trees

#13  "mental fatigue"

#14  "mood disorder*"

#15  "affective disorder*"

#16  #1or#2or#3or#4or#5or#6or#7or#8or#9or#10or#11or#12or#13or#14or#15

#17  MeSH descriptor: [Ketogenic Diet] this term only

#18  "ketogenic diet"

#19  MeSH descriptor: [Diet, Paleolithic] this term only

#20  "paleolithic diet" or "palaeolithic diet" or "paleo diet" or "caveman diet"

#21  MeSH descriptor: [Diet, Carbohydrate-Restricted] this term only

#22  "carbohydrate restricted diet" or "carbohydrate-restricted diet"

#23  "low carbohydrate high fat diet"

#24  "low carbohydrate diet"

#25  "high fat diet"

#26  "high fat low carbohydrate diet"

#27  "lchf diet" or "vlchf diet" or "lckd diet" or "hflc diet"

#28  #17or#18or#19or#20or#21or#22or#23or#24or#25or#26or#27

#29  #16 and #28

**3. Database: Ovid MEDLINE(R) In-Process & Other Non-Indexed Citations**

Search Strategy:
--------------------------------------------------------------------------------
1 (depression or depressed or depressive).mp. [mp=title, abstract, original title, name of substance word, subject heading word, keyword heading word, protocol supplementary concept word, rare disease supplementary concept word, unique identifier]

2 (anxiety or anxious).mp. [mp=title, abstract, original title, name of substance word, subject heading word, keyword heading word, protocol supplementary concept word, rare disease supplementary concept word, unique identifier]

3 dysthymia.mp.
4 dysthymic disorder.mp.

5  low mood.mp.

6  mental fatigue.mp.

7  mood disorder$.mp.

8  affective disorder$.mp.

9  1or2or3or4or5or6or7or8

10  dietary carbohydrate$.mp.

11  ((intake or diet$1) adj2 carbohydrate$).mp.

12  10 or 11

13  ((high or rich) adj2 carbohydrate$).mp.

14  12 and 13

15  high carb diet$.mp.

16  14 or 15

17  low carbohydrate high fat diet$.mp.

18  16 not 17

19  (high gly?emic index adj2 diet$).mp. [mp=title, abstract, original title, name of substance word, subject heading word, keyword heading word, protocol supplementary concept word, rare disease supplementary concept word, unique identifier]

20  18 or 19

21  9 and 20

22  from 21 keep 2-4

23  21 not 22

**4. Database: Ovid MEDLINE(R), Embase, PsycINFO**

Search Strategy: --------------------------------------------------------------------------------

1  Ketogenic Diet/

2  Ketogenic Diet.mp.

3  Diet, Paleolithic/

4  paleolithic diet/

5  paleolithic diet.mp.

6  palaeolithic diet.mp.

7  paleo diet.mp.

8  caveman diet.mp.

9  (stone-age diet or stone age diet).mp. [mp=ti, ab, ot, nm, hw, kf, px, rx, ui, tn, dm, mf, dv, kw, tc, id, tm]

10  Diet, Carbohydrate-Restricted/

11  low carbohydrate diet/

12  carbohydrate-restricted diet.mp.

13  carbohydrate restricted diet.mp.

14  low carbohydrate diet.mp.

15  low carbohydrate high fat diet$.mp.

16  high fat diet.mp.

17  lchf diet.mp.

18  vlchf diet.mp.

19  lckd diet.mp.

20  hflc diet.mp.

21  high fat low carbohydrate diet.mp.

22  or/1-21

23  Bipolar Disorder/

24  exp bipolar disorder/

25  (bipolar or manic depressive or manic depression).mp. [mp=ti, ab, ot, nm, hw, kf, px, rx, ui, an, tn, dm, mf, dv, kw, tc, id, tm]

26  schizoaffective psychosis/

27  schizoaffective disorder/

28  schizoaffective psychosis.mp.

29  schizoaffective disorder.mp.

30  schizo affective psychosis.mp. [mp=ti, ab, ot, nm, hw, kf, px, rx, ui, an, tn, dm, mf, dv, kw, tc, id, tm]

31  schizo affective disorder.mp.

32  or/23-31

33  22 and 32

34  remove duplicates from 33

35  limit 34 to humans [Limit not valid in PsycINFO; records were retained]

36  (mice or mouse or rat or animal).mp.

37  35 not 36

**5. Database: Ovid MEDLINE(R) In-Process & Other Non-Indexed Citations, Ovid MEDLINE(R) Epub Ahead of Print**

Search Strategy: --------------------------------------------------------------------------------

1  Ketogenic Diet.mp.

2  paleolithic diet.mp.

3  palaeolithic diet.mp.

4  paleo diet.mp.

5  caveman diet.mp.

6  (stone-age diet or stone age diet).mp. [mp=title, abstract, original title, name of substance word, subject heading word, keyword heading word, protocol supplementary concept word, rare disease supplementary concept word, unique identifier]

7  carbohydrate-restricted diet.mp.

8  carbohydrate restricted diet.mp.

9  low carbohydrate diet.mp.

10  low carbohydrate high fat diet$.mp.

11  high fat diet.mp.

12  lchf diet.mp.

13  vlchf diet.mp.

14  lckd diet.mp.

15  hflc diet.mp.

16  high fat low carbohydrate diet.mp.

17  (bipolar or manic depressive or manic depression).mp. [mp=title, abstract, original title, name of substance word, subject heading word, keyword heading word, protocol supplementary concept word, rare disease supplementary concept word, unique identifier]

18  schizoaffective psychosis.mp.

19  schizoaffective disorder.mp.

20 schizo affective psychosis.mp. [mp=title, abstract, original title, name of substance word, subject heading word, keyword heading word, protocol supplementary concept word, rare disease supplementary concept word, unique identifier]

21 schizo affective disorder.mp.

22 1or2or3or4or5or6or7or8or9or10or11or12or13or14or15or16

23 17or18or19or20or21

24 22 and 23

**6. Search Name: Ketogenic diet and bipolar or schizoaffective Description: Cochrane** ID Search Hits

#1  MeSH descriptor: [Ketogenic Diet] this term only

#2  "ketogenic diet" .mp

#3  MeSH descriptor: [Diet, Paleolithic] this term only

#4  "paleolithic diet" or "palaeolithic diet" or "paleo diet" or "caveman diet"

#5  MeSH descriptor: [Diet, Carbohydrate-Restricted] this term only

#6  "carbohydrate restricted diet" or "carbohydrate-restricted diet"

#7  "low carbohydrate high fat diet"

#8  "low carbohydrate diet"

#9  "high fat diet"

#10  "high fat low carbohydrate diet"

#11  "lchf diet" or "vlchf diet" or "lckd diet" or "hflc diet"

#12  #1 or #2 or #3 or #4 or #5 or #6 or #7 or #8 or #9 or #10 or #11

#13  MeSH descriptor: [Bipolar and Related Disorders] explode all trees

#14  bipolar or schizoaffective or "schizo affective"

#15  #13 or #14

#16  #12 and #15
