# Supplementary material for: Efficacy of low carbohydrate and ketogenic diets in treating mood and anxiety disorders: systematic review and implications for clinical practice
Source: BJPsych Open. 2023 Apr 17;9(3):e70. doi: 10.1192/bjo.2023.36 (PMC10134254; doi:10.1192/bjo.2023.36)
Supplement: Supplementary file 1 [file bjosup.zip › S2056472423000364sup002.docx]

**Supplementary file 2 - Risk of bias assessment**

| **Study** | **Selection/Recruitment** | **Assessment of Exposure** | **Assessment of Confounders** | **Assessment of Outcome(s)** |
| --- | --- | --- | --- | --- |
| Campbell & Campbell 2019 | -  Participants were self-selected, participating on online forums. Unlikely to be representative. | -  Diet exposure and plausibility of achieving ketosis was assessed via self-report. | -  None assessed | **-**  Symptom responses to diet was assessed via retrospective self-report only |
| Danan et al (2022) | -  Psychiatric inpatients; volunteers selected by the author. No non-exposed comparison group. | +  Diet provided to participants in the hospital, adherence monitored via physician interview, food journals, and nursing observation. Ketosis assessed via urinalysis. | -  Metabolic health outcomes were measured, but not considered as potential confounders. | **+**  Psychiatric symptoms assessed via validated questionnaires and clinician interview measures. |
| Kunin (1976) | -  Psychiatric outpatients; recruitment not specified in detail. No non-exposed comparison group. | +  Diet instructions provided and participants tracked their intake in food logs. Ketosis assessed via urinalysis. | -  None assessed | -  Symptom responses to diet were self-reported; assessment tool not specified. |
